# The conservation physiology toolbox: status and opportunities

**DOI:** 10.1093/conphys/coy029

**Published:** 2018-06-19

**Authors:** Christine L Madliger, Oliver P Love, Kevin R Hultine, Steven J Cooke

**Affiliations:** 1 Fish Ecology and Conservation Physiology Laboratory, Department of Biology and Institute of Environmental Science, Carleton University, 1125 Colonel By Dr., Ottawa, Ontario, Canada; 2 Department of Biological Sciences, University of Windsor, 401 Sunset Ave., Ontario, Canada; 3 Department of Research, Conservation and Collections, Desert Botanical Garden, 1201 N. Galvin Parkway, Phoenix, AZ, USA

**Keywords:** Tools, techniques, glucocorticoid, validation, biomarker

## Abstract

For over a century, physiological tools and techniques have been allowing researchers to characterize how organisms respond to changes in their natural environment and how they interact with human activities or infrastructure. Over time, many of these techniques have become part of the conservation physiology toolbox, which is used to monitor, predict, conserve, and restore plant and animal populations under threat. Here, we provide a summary of the tools that currently comprise the conservation physiology toolbox. By assessing patterns in articles that have been published in ‘Conservation Physiology’ over the past 5 years that focus on introducing, refining and validating tools, we provide an overview of where researchers are placing emphasis in terms of taxa and physiological sub-disciplines. Although there is certainly diversity across the toolbox, metrics of stress physiology (particularly glucocorticoids) and studies focusing on mammals have garnered the greatest attention, with both comprising the majority of publications (>45%). We also summarize the types of validations that are actively being completed, including those related to logistics (sample collection, storage and processing), interpretation of variation in physiological traits and relevance for conservation science. Finally, we provide recommendations for future tool refinement, with suggestions for: (i) improving our understanding of the applicability of glucocorticoid physiology; (ii) linking multiple physiological and non-physiological tools; (iii) establishing a framework for plant conservation physiology; (iv) assessing links between environmental disturbance, physiology and fitness; (v) appreciating opportunities for validations in under-represented taxa; and (vi) emphasizing tool validation as a core component of research programmes. Overall, we are confident that conservation physiology will continue to increase its applicability to more taxa, develop more non-invasive techniques, delineate where limitations exist, and identify the contexts necessary for interpretation in captivity and the wild.

## Introduction

Conservation physiology aims to apply an array of physiological concepts, tools and techniques to characterize biodiversity and predict multi-scale responses to environmental change. The field’s deepest roots lie in plant physiology, which traces back to experiments on growth completed in the early 1600s (Egerton, 2012). Nearly a century later in the early-to-mid 1700s, plant physiology began to solidify as a distinct discipline when transpiration, growth and water uptake were becoming better characterized (Egerton, 2012). As the study of plant physiology continued to progress in the early nineteenth century and began quantifying the importance of inorganic nutrients to plant growth (Pennazio, 2005), comparative animal physiology was beginning to take shape by characterizing the physiological functioning of animals in the laboratory (reviewed in Feder *et al.*, 1987). Ecological physiology, which represents a merging of comparative physiology and natural history, began to develop as scientists gained greater interest in how an organism’s physiology allows it to respond to natural environmental conditions (Costa and Sinervo, 2004). By the late 1940s, pioneers in the field of animal ecological physiology were determining the physiological mechanisms underpinning diving, water and temperature balance in xeric desert environments, and tolerance and temperature regulation in polar regions (Feder *et al.*, 1987). In parallel, the ecological physiology of plants gained significant steam with the advent of gas analyzers and stable isotope techniques that broadened researchers’ abilities to study resource acquisition, inter-specific interactions, the function of plants in a given ecosystem and carbon and water fluxes (Dawson *et al.*, 2002). Ecophysiology has now grown from primarily studying basic physiological processes and the physiological adaptations of organisms to extreme environments to a highly interdisciplinary field that combines behavioural ecology, life history theory, molecular biology and evolutionary biology to characterize physiology across all types of environmental conditions and spatial and temporal scales (Willmer *et al.*, 2009). All of this fascinating growth in scope has led to rapid advancements in field physiology and our ability to quantify physiological traits across environments in natural settings (Costa and Sinervo, 2004). Today, every facet of ecological physiology has influenced the development of conservation physiology.

At their core, conservation physiology techniques are decision-support tools. The ultimate goal of the field is to enable initiatives with positive outcomes for species of concern (whether imperiled or as part of sustainable management) based on the collection and interpretation of physiological data (Cooke *et al.*, 2013b). With this aim comes a responsibility to validate the accuracy, precision and context-dependency of the techniques we use to ensure that conservation decisions are as well-informed as possible. When the field of conservation physiology was formally outlined in 2006, Wikelski and Cooke suggested that eight physiological sub-disciplines could contribute valuable tools for conservation science—endocrinology, environmental physiology, comparative physiology, evolutionary physiology, immunology/epidemiology, physiological genomics, neurophysiology and toxicology. Since then, this number has been refined and grown by one third, encouraging directed tool development in diverse topic areas that also include bioenergetics, cardiorespiratory physiology, reproductive physiology and many others (Cooke *et al.*, 2013b). Here, we provide a brief outline of the current breadth of the conservation physiology toolbox. We also include an overview of papers that have been published in ‘Conservation Physiology’ (January 2013–January 2018) that specifically focus on techniques and methods that will improve our ability to use existing tools or add new ones to our arsenal. By doing so, we indicate where researchers have been focusing their effort in terms of physiological variables and taxa, the specific ways that they have been contributing to tool refinement, and ways to promote beneficial growth in the future. From the outset we acknowledge that plants have received comparatively less attention than animals in conservation physiology studies. As such, any apparent bias towards animals is simply a reflection of that, and we make specific reference to needs and opportunities for plants in the concluding section of the paper to help address this deficiency.

## What do we have in the toolbox?

While numerous physiological tools at our disposal have been measured in the wild for decades, many are now being actively validated for the express purpose of applying them to conservation challenges. We provide a list of the tools and techniques that currently comprise the conservation physiology toolbox, their associated physiological sub-discipline, and examples of their applications within the field of conservation physiology in Table 1. The toolbox is noticeably diverse in a number of ways. First, physiological variables can now be measured in a highly varied array of sample media including blood, outer integuments (e.g. fur, feathers), tissue biopsies, saliva, eggs, urine and faeces, respired air and water (e.g. hormones released via gills). Because of this variety of sample types, the time period for which physiological information is available also varies. Tissues like whale baleen can provide a profile of changing hormone concentrations over the course of decades (Hunt *et al.*, 2014), while blood samples provide a snapshot of circulating hormone levels at the time the sample was obtained (Romero and Reed, 2005; Lawrence *et al.*, 2018). This diversity of sample media also translates into differences in invasiveness and duration of handling required, ranging from mildly invasive (e.g. blood drawing) to low invasiveness (e.g. obtaining a hair or feather sample) to non-invasive (e.g. passive faeces collection).

**Table 1:** List of physiological tools currently found in the conservation physiology toolbox, their associated physiological sub-discipline, and examples of their contributions to conservation science

Physiological sub-discipline	Tool	Examples of roles in conservation science
Bioenergetics and nutritional physiology	Amino acid profiles	Assessing whole-organism response to environmental change; improving captive breeding and rehabilitation through adequate nutrition; monitoring conservation management scenarios; identifying mechanisms behind population decline
	Body composition	
	Body condition	
	Body temperature	
	Energy expenditure	
	Lipid and fatty acid concentrations	
	Metabolic rate	
	Nutritional state/deficiency	
	Other plasma metabolites (e.g. beta-hydroxy butyrate)	
	Plasma glucose	
	Plasma lactate	
	Stable isotopes	
Cardiorespiratory physiology	Aerobic scope	Predicting responses to environmental change; predicting invasive species spread; predicting species distributions under climate change scenarios
	Carbon dioxide partial pressure	
	Haematocrit	
	Haemoglobin concentration	
	Heart rate	
	Muscle enzymes (e.g. citrate synthase, lactate dehydrogenase)	
	Muscle oxygenation	
	Myoglobin concentration	
	Oxygen partial pressure	
	Respiratory rate	
Chemical communication and non-stress/non-reproductive endocrine measures	Aldosterone	Determining influence of environmental change on growth and development; allowing better interpretation of stress physiology
	Growth hormone	
	Melatonin	
	Plant growth regulators (i.e. plant hormones)	
Environmental plant physiology	Growth and development	Understanding plant responses to environmental change; improving restoration success
	Gas exchange (respiration, photosynthesis, stomatal conductance, transpiration)	
	Leaf structure (specific leaf area, leaf size and shape)	
	Nutrient sources, pathways and transport	
	Plant hydraulics (xylem cavitation vulnerability, xylem hydraulic conductivity, hydraulic architecture)	
	Phenology	
	Stable isotopes (indicators of stress, photosynthetic pathway, water and nitrogen sources)	
Immunology/epidemiology	Disease state (e.g. serum total protein)	Predicting spread of diseases; improving design of control and vaccination programmes; determining sub-lethal consequences of environmental change
	Humoral and cell-mediated immune response	
	Microbiomes (e.g. gut, respiratory, epidermal)	
Neurophysiology/sensory biology	Neural activity	Determining guidelines/optimal designs to reduce human-wildlife conflict; understanding mechanisms behind behavioural responses to environmental change
	Neuropeptides	
	Pheromones	
	Sensory sensitivities and tolerances	
Physiological genomics and proteomics	Gene arrays	Quantifying molecular physiological diversity; understanding multi-faceted responses to environmental change
	Protein microarrays	
Plant carbon balance/stress tolerance	Chlorophyll fluorescence (electron transport capacity of Photosystem II)	Identifying plant tolerance to extreme thermal stress, drought, episodic disturbance and pollution
	Non-structural carbohydrate concentration	
	Leaf temperature (photosynthetic enzyme activity and efficiency)	
Reproductive physiology	Oxytocin	Identifying mechanisms behind population declines; improving captive breeding success; monitoring success of reintroduction programmes
	Pregnancy rate	
	Reproductive hormones (e.g. estrogen, testosterone, progesterone)	
	Sperm physiology	
	Vitellogenin	
Stress physiology	Glucocorticoids	Predicting and monitoring responses to environmental change; monitoring success of conservation programmes
	Heat shock proteins	
	Heterophil to lymphocyte ratio	
	Oxalate	
	Oxidative status	
	Pentosidine	
	pH (e.g. gastric, blood)	
	Plant stress response (e.g. anthocyanins)	
	Plasma ion concentration (e.g. sodium, chloride, potassium)	
	Resistance (e.g. pH, salinity, desiccation, inundation)	
	Telomeres	
	Thermal tolerance (e.g. CTMax)	
Toxicology	Pollutant/chemical contaminant concentrations	Determining sources of population declines; delineating regulatory guidelines; designing remediation protocols
	Trace element/metal concentrations	

Further related to logistics, there are large differences in the cost of the various physiological techniques in the toolbox. Some, like many hormones and various ‘omic’ approaches, can require expensive laboratory equipment along with a moderate cost output for each sample analysed. At the other extreme, indices of body condition can have little to no monetary cost to collect, apart from accessing the organism in the field. A final source of diversity related to logistics is the speed with which physiological information can be obtained following sample collection. Some techniques require that samples be stored, shipped and analysed in a laboratory setting which may require days to months to be fully processed [e.g. feather glucocorticoids (GCs) from samples collected at a remote field site], others require handling of animals for extended time periods (e.g. respirometry to obtain metabolic rate), while others may be procured almost instantaneously (e.g. blood samples analysed with point-of-care devices for plasma glucose and lactate). The vast datasets generated by some techniques (especially ‘omics’ and biologging/telemetry) can also require sophisticated data analyses that further extend the time course of generating conclusions. Given this range of options, and the fervor with which researchers are now working to decrease the invasiveness and increase the efficiency of measuring many components of physiology (e.g. stress physiology: Kersey and Dehnhard, 2014; bioenergetics: Mochnacz *et al.*, 2017), the conservation physiology toolbox is continuing to grow more accessible from a logistical stand-point.

The current toolbox also reflects the variety of biological scales from which physiological measurements can be obtained. Researchers are investigating how organisms respond to and interact with their environment at the level of gene expression (e.g. physiological transcriptomics), gene products (e.g. physiological proteomics), cells (e.g. sperm physiology), individual tissues (e.g. muscle oxygenation), organs (e.g. heart rate) and the whole-organism (e.g. daily energy expenditure). As a result, different components of physiology are better-suited to certain conservation goals (Table 1). For example, reproductive physiological parameters can be highly relevant for designing and improving captive breeding programmes (e.g. Dehnhard *et al.*, 2008), while aspects of cardiorespiratory physiology (e.g. aerobic scope) have been useful for predicting the tolerance and spread of invasive species (e.g. Marras *et al.*, 2015).

Researchers and practitioners must weigh the costs and benefits in terms of all of the aforementioned characteristics when deciding whether a given physiological tool is applicable in their system, with some tools being more established or validated than others. However, the novelty of a tool does not necessarily indicate how much validation is required. For example, GCs have been measured in wild vertebrates for decades (Wingfield and Farner, 1976), but they are still being actively validated across multiple sample media types to quantify stability (e.g. feather corticosterone: Romero and Fairhurst, 2016; faecal GCs: Wilkening *et al.*, 2016), extraction efficiency (e.g. faecal GCs: Hayward *et al.*, 2010), effects of metabolic rate and diet (faecal GCs: Goymann, 2012), and correlation with environmental disturbance and fitness (e.g. plasma GCs: Breuner *et al.*, 2008; Bonier *et al.*, 2009; Madliger and Love, 2016; faecal GCs: Lea *et al.*, 2018). Alternatively, other variables have been measured by ecological physiologists for decades, but are relatively new in terms of their application to conservation science (e.g. immune function: Norris and Evans, 2000; Hing *et al.*, 2016; Becker *et al.*, 2018). As a result, they require validations regarding their feasibility for use in this novel context, such as their relationship to the demographic parameters that drive population responses to environmental change (e.g. oxidative status: Beaulieu and Costantini, 2014; immune function: Pedersen and Babayan, 2011). Other well-recognized physiological variables are being physically measured in novel ways (e.g. plasma glucose via point-of-care devices) and need validation in this new format to ensure their accuracy in non-human systems (Stoot *et al.*, 2014). Finally, some tools are so new to the field that most validation is still to come (e.g. pentosidine as a biomarker of ageing in ectotherms: Iverson *et al.*, 2017). Overall, the types of validations that are needed vary as a result of these patterns; they may be related to logistics (collection, storage, preparation, quantification), interpretation or both (see detailed discussion below).

## Where are researchers placing emphasis for tool development?

Conservation Physiology’s ‘Tool Box’ papers focus on techniques or methods that will improve our ability to use existing tools or add new ones to our arsenal. The techniques presented in these articles can be lab- or field-based, and since the launch of the journal in 2013, 23 Tool Box articles have been published. To examine where researchers are focusing validation efforts, we assessed the papers in the Tool Box section as well as other Conservation Physiology articles (i.e. standard research articles) that indicated they are specifically completing a validation, or introducing, investigating the utility of, or refining a tool or technique. We identified validation-focused articles that were not published under the Tool Box heading by searching the journal’s archives using the search string (valid* OR tool OR technique OR novel), and then checking each result to confirm it contained a validation, refinement or introduction of a novel methodology. Overall, we assessed patterns across 73 articles published from the launch of the journal in January 2013 through January of 2018 (Supplementary Table S1).

**Figure 1:**
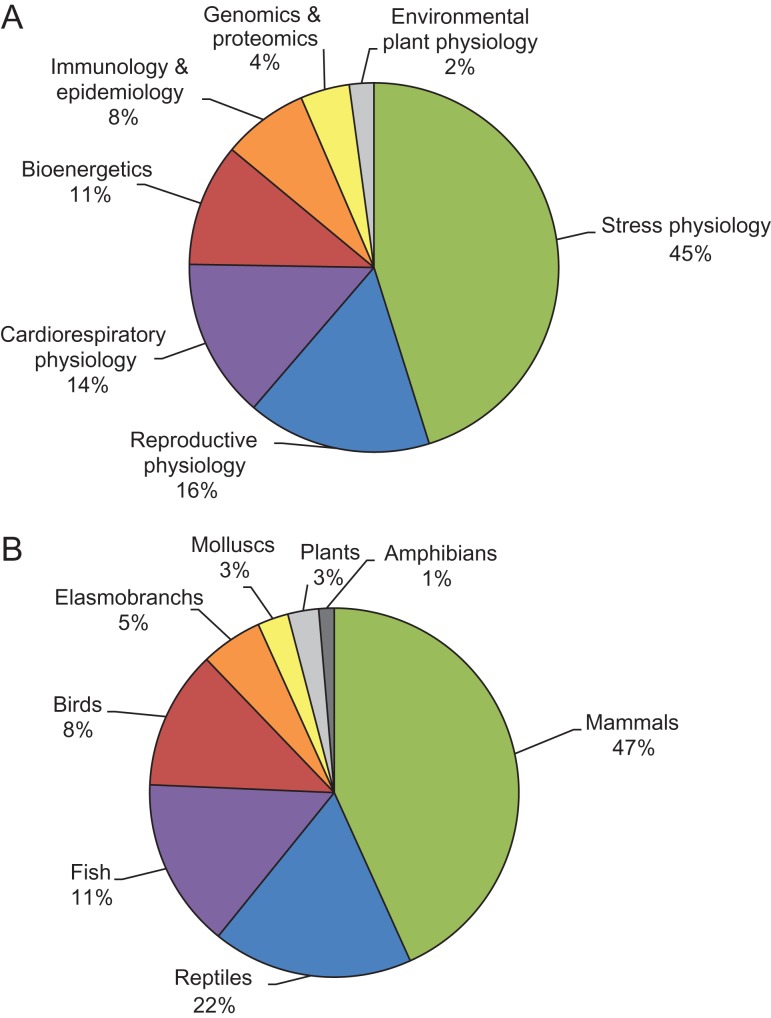
Focus of the 73 tool-based (validation or proposal of novel technique) papers published in ‘Conservation Physiology’ in the past 5 years (2013–18) based on: (A) physiological sub-discipline and (B) taxonomic group. Note: Fish includes all bony and cartilaginous fish aside from those that are elasmobranchs, which are featured as a separate category

While the overall diversity of topics is to be praised, bias remains with regards to which sub-disciplines have garnered the greatest attention (Fig. 1A). Over half of the articles were focused on hormones (stress and reproductive endocrinology), and many of these on various non-invasive methods of GC measurement in faeces, hair, feathers and other integuments. In some cases, the focus is on obtaining GCs from novel tissues. For example, Bechshoft *et al.* (2015) developed a technique to sample, extract and quantify cortisol levels in skin samples from harbour porpoises (*Phocoena phocoena*) using gas chromatography–tandem mass spectrometry. In other cases, researchers have developed novel methodologies for obtaining samples less invasively. To this end, Newman *et al.* (2015) successfully obtained cortisol measurements via water sampling from Atlantic salmon (*Salmo salar L.*) fry at the dispersal stage, an age class previously only sampled by sacrificing fish to obtain plasma or whole body GC measurements. Finally, others have performed validations on well-established methods to increase our ability to interpret GC concentrations accurately. For example, Berk *et al.* (2016) drew attention to a number of methodological considerations regarding corticosterone measurement in feathers, citing issues with small samples containing unexpectedly high hormone levels and cross-reactivity with other hormone metabolites. Overall, the measurement of GCs in conservation settings is still a very active arena of tool development for conservation physiologists, despite being a commonly measured metric of stress physiology in general.

It is also apparent that some tools have been relatively under-represented in the journal. For example, none of the validation papers focused on toxicology. In this case, it may be that the tools associated with this sub-discipline have been developing for use in conservation science for decades. Indeed, the toxicological effects of DDT on avian reproductive physiology is often cited as one of the earliest and clearest successes of the conservation physiology approach (Wikelski and Cooke, 2006). Genomics, transcriptomics and proteomics also did not make up a large portion of the validation articles (4%). However, in this case, these tools are relatively new to conservation physiology and are receiving ample attention, especially since high-throughput technologies have become established (Kohn *et al.*, 2006). As a result, it is more likely that the validations of these techniques are being published in other venues focused on conservation genetics (which represents its own arm of conservation science) or journals focused on molecular biology and genetics (e.g. BMC Genomics: Campana et al., 2016; Kleinman-Ruiz et al., 2017; Conservation Genetics Resources: Shi et al., 2018; Molecular Ecology Resources: Hess et al., 2015; Molecular Ecology: Moore *et al.*, 2014). Nonetheless, these techniques are extremely valuable to the field of conservation physiology, providing insight into a fundamental level of biodiversity and how it should be conserved, and which parts of the genome contribute most to a given species’ survival (McMahon *et al.*, 2014). It is evident from the growth of genomics within the context of conservation science (Kohn *et al.*, 2006; Allendorf *et al.*, 2010; Ouborg *et al.*, 2010; McMahon *et al.*, 2014; Garner *et al.*, 2016) that the entire suite of ‘-omics tools’ will provide opportunities to generate links between gene and protein expression patterns and specific environmental stressors to improve the suite of conservation physiology biomarkers at our disposal. Molecular methods are also increasingly being applied for aspects of pathogen surveillance, and when linked to physiological status, provide mechanistic understanding of factors influencing organism health and survival (Teffer *et al.*, 2017).

The techniques of focus across validations have also pertained to a variety of taxa including mammals, fish, elasmobranchs, reptiles, birds, amphibians, plants and molluscs (Fig. 1B). However, very few investigations have included invertebrates, plants or amphibians as the study species (<3% in all cases), despite these taxa being highly relevant in regards to the global biodiversity crisis (IUCN, 2017). In fact, nearly half of the articles published focus on mammals. Some of this taxonomic imbalance may be a reflection of a conservation-based funding or research-interest bias towards large, charismatic mammals (Czech *et al.*, 1998; Clark and May, 2002). Further, the body sizes of many of the mammals studied (e.g. bears, whales, seals, tigers) offer the ability to retrieve relatively large tissue or faecal samples, compared to many smaller fish, birds, and amphibians. We do not believe that the taxonomic bias is likely due to physiological tools needing more validation in mammalian organisms. Indeed, many physiological assays and technologies are adapted from use in humans, leading to the assumption that their application to mammals should be more straightforward.

Another theme that can be gleaned from an assessment of the journal’s tool-based papers is the rising use of rapid field assessment technologies. These hand-held devices are appealing because they eliminate the need for storage and transport of samples, provide immediate results, are highly portable, and are low-cost compared to many laboratory procedures (Stoot *et al.*, 2014). Point-of-care devices can measure a variety of physiological variables including haemoglobin concentration and blood glucose, lactate and pH (Stoot *et al.*, 2014). However, because these devices were designed for the analysis of human blood, it is still necessary to conduct species-specific calibrations and validations to ensure accuracy in the wild (Stoot *et al.*, 2014), and a handful of investigations with this focus were included among the tool-based articles published over the past 5 years. For example, Harter *et al.* (2015) confirmed that the i-STAT portable clinical analyser was able to obtain accurate measures of blood pH in the sandbar shark (*Carcharhinus plumbeus*), but could not reliably indicate partial pressure of oxygen or haemoglobin oxygen saturation measurements. Likewise, when Bennett *et al.* (2017) compared laboratory testing of blood glucose with measurements obtained from a portable glucometer in wild grey seals (*Halichoerus grypus*), they found the glucometer to be highly precise, but not accurate. While these validations indicate true limitations for use in some species, there are circumstances where adjustments can be made to allow for biologically accurate results. For example, Andrewartha *et al.* (2016) found that while the HemoCue point-of-care device overestimated haemoglobin concentration in both blue-tongued skinks (*Tiliqua nigrolutea*) and Atlantic salmon (*Salmo salar*), calibration equations can rectify this issue. Overall, we anticipate continued interest in point-of-care devices over the coming years, and we believe the studies accumulating in ‘Conservation Physiology’ can provide excellent road maps for completing the necessary intra- and inter-specific tool validations.

**Figure 2:**
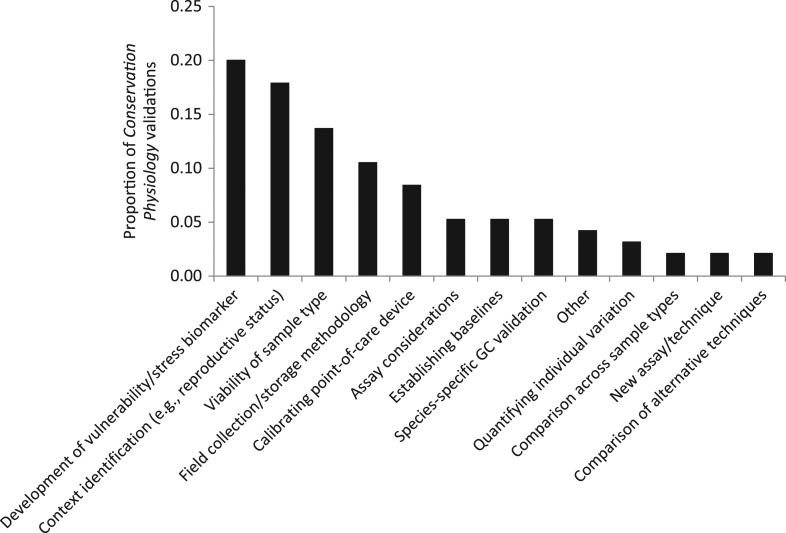
Types of validations completed in the 73 tool-based papers published in ‘Conservation Physiology’ in the past 5 years (2013–18). Some articles included more than one type of validation

A final theme we observed across tool-based publications is that researchers have placed emphasis on validating tools for a variety of reasons, including sample collection, storage, analysis and interpretation (Fig. 2). The largest proportion of validations focused on identifying physiological biomarkers that can indicate vulnerability (e.g. to disease or mortality), stress level or response to a habitat disturbance. For example, Ellenberg *et al.* (2013) assessed whether heart rate could provide a measure of disturbance caused by human presence in yellow-eyed penguins (*Megadyptes antipodes*). Interestingly, they found that the birds assessed the level of disturbance (i.e. threat) differently than they had predicted, indicating that this type of physiological measure could provide an objective and unprecedented assessment of disturbance in this species. Another important validation for any tool proposed as a potential biomarker of disturbance is to link variation in the physiological trait to population-level processes, thus allowing researchers to predict how populations could change based on measurements from individuals (Cooke and O’Connor, 2010). For example, Costantini and Dell’Omo (2015) confirmed in Scopoli’s shearwater (*Calnectris diomedea*) that a component of oxidative stress (reactive oxygen metabolites) was related to reproductive success and survival probability. In contrast, other metrics of oxidative stress (non-enzymatic antioxidant capacity of plasma and thiols) did not predict any components of fitness, providing information that defines how this tool can be used as a biomarker to predict population change. Clearly, more work is needed on refinement of oxidative stress indicators and status for conservation science (Beaulieu and Costantini, 2014), including how different measurement techniques compare and perform (Monaghan *et al.*, 2009; Hõrak and Cohen, 2010), the ecological factors that influence oxidative stress (Monaghan *et al.*, 2009; Costantini *et al.*, 2010), and the level of intra-specific variability in how this component of physiology responds to environmental perturbations (Costantini *et al.*, 2010).

Another substantial proportion of articles focused on identifying whether a physiological metric reflected characteristics (e.g. sex, reproductive status, season, year) of the organism being sampled (Fig. 2). In some cases, the ultimate goal was to use the tool to identify the status of an animal. For example, Polegato *et al.* (2018) determined that faecal samples analysed for progestins reflect changing phases of the reproductive cycle in captive marsh deer (*Blastocerus dichotomus*), indicating this technique can be used to identify reproductive status in this species. In other cases, the validation determined which intrinsic contexts need to be considered when interpreting physiological values. Di Francesco *et al.* (2017) found that cortisol concentration in Muskoxen (*Ovibos moschatus*) hair varied based on sex, season and year, illustrating the importance of considering multiple contexts when monitoring GC levels through time.

The validation of tissue types as viable samples for physiological assay and improving collection and storage of existing sample types from the field were also common investigations (Fig. 2). By comparing sample age and exposure to sun and water, Wong *et al.* (2016) determined that faecal GC metabolites in Asian elephant (*Elaphas maximus*) dung will remain stable in tropical environments for up to 8 hours, providing guidance regarding sample stability in the wild. In other cases, entirely new tools have been introduced. Carlson *et al.* (2016) developed a novel anti-body based protein microarray that could assess the expression of 31 stress-related proteins simultaneously from a small skin biopsy (similar to what could be obtained from a remotely deployed biopsy dart) in grizzly bears (*Ursus arctos*). Further, some tool-focused papers calibrated certain techniques in a species- or taxa-specific manner. Flint *et al.* (2015) used healthy Indo-Pacific green sea turtles (*Chelonia mydas*) to determine plasma protein reference intervals for electrophoresis, and compared these to profiles from unhealthy turtles being cared for at a wildlife hospital. By establishing these baselines, this technique can better be used to identify disease status in endangered sea turtles. Additionally, other validations focused on taking tools that are well-established in one taxa and optimizing them for use in a different group of organisms. Focusing on a phytohaemagglutinin inflammation assay that is well-validated in birds, Clulow *et al.* (2015) validated and optimized the assay for use in amphibians, broadening the ecoimmunological toolbox for this group.

Finally, researchers have also drawn attention to existing short-comings with some tools, allowing for directed future study that can refine techniques to delineate their limitations within the context of conservation settings. Lafferty *et al.* (2017) assessed sample size requirements for faecal GC metabolite concentrations in snowshoe hares (*Lepus americanus*) to detect population-level variation, finding that small effect sizes could lead to this technique being cost-prohibitive or not logistically feasible in certain species. Building off previous work, Drake *et al.* (2017) found that classic blood diagnostics were not providing sufficient information to determine health in the threatened Mojave desert tortoise (*Gopherus agassizii*). However, combining this diagnostic panel with gene transcription profiling provided a better measurement of health and immune function (Drake *et al.*, 2017). It is likely that combining multiple physiological measures will benefit our understanding of the consequences of anthropogenic change in many organisms (Madliger and Love, 2015).

## Next steps: continuing to improve the value of the toolbox

We acknowledge that our assessment of tool development was limited to a single journal. However, it is the only discipline-specific journal for the field and this cross-section therefore allowed us to focus on directed tool development specifically for conservation physiologists. Given the strong first Impact Factor (from Clarivate Analytics) recently calculated in late 2016 (2.32), it is clear that the journal’s publications are contributing to the ever-quickening growth of the field. For these reasons, we are confident that our sample of publications provides insight into current trends to help identify areas for further growth. Here, we present a number of suggestions for pressing forward.

### Assess the conservation value of measuring GCs

Metrics of stress physiology are among the most commonly used tools in conservation physiology, and GCs in particular continue to amass popularity as indicators of disturbance in wildlife (Dantzer *et al.*, 2014; Lennox and Cooke, 2014; Sopinka *et al.*, 2015). The desire to ascertain physiological stress through GC concentrations is evidenced by the array of novel sample types continually being investigated, which has recently extended to whale ear wax (Trumble *et al.*, 2013) and amphibian skin secretions (Santymire *et al.*, 2018). If a sample type could potentially contain GCs, researchers are working to collect, process and assay it as effectively as possible, and we applaud the ingenuity involved. However, given the staggering literature measuring GCs in wildlife, the applications and limitations for conservation can be difficult to demarcate. As a result, we are in need of meta-analyses that can summarize the available research, specifically interpreting patterns to identify the optimal circumstances for GC application (e.g. Dantzer *et al.* 2014). Namely, we need to determine whether specific taxa, sample types, seasons, developmental or life history stages, disturbance types or life history strategies (e.g. semelparous vs. iteroparous) are more likely to show predictable, biologically relevant (i.e. disturbance and fitness-related) changes in GC concentrations. Further, documented examples of GC measurements influencing conservation success (i.e. on-the-ground management) need to be identified and quantified. For example, it would be highly informative to know how often GC measurements have identified populations in need of management intervention or have been used to monitor the realized success of restoration activities. This is not trivial, as much of this information is not reported in the primary literature; however, it is crucial for determining whether the enormous effort placed on quantifying GC levels is benefiting conservation.

An additional profitable future endeavour will involve the explicit comparison of GCs with other metrics available for conservation monitoring. Over 10 years ago, Tarlow and Blumstein (2007) performed a non-quantitative investigation that compared seven metrics of potential anthropogenic stress in animals (breeding success, cardiac response, flight initiation distance, fluctuating asymmetry, GCs, immunocompetence and mate choice), assigning each a relative rating of high, medium or low in terms of ease of use, ability to quantify impact, reflection of population viability and repeatability. GCs scored a ranking of medium in all categories. However, given the torrent of investigations involving GCs since the formal description of conservation physiology and the even greater literature base that has accumulated in eco- and evolutionary physiology since the Cort-Fitness Hypothesis was formalized (Bonier *et al.*, 2009), there currently exists the possibility to compare metrics using a quantitative, meta-analytic framework (e.g. Sorenson *et al.*, 2017). In particular, estimates of cost, time investment, sample storage requirements and invasiveness are warranted, as well as the inclusion of other physiological and behavioural metrics. Overall, comparisons among metrics will help practitioners weigh the costs and benefits of alternative techniques for their wildlife systems.

### Combine multiple physiological and non-physiological tools

In circumstances where a conservation physiology tool faces a limitation, it may be possible to combine it with other physiological or non-physiological measures to allow for improved interpretations. For example, faecal GCs can show a high level of variation due to sex and life history stage in North Atlantic right whales (*Eubalaena glacialis*), making it more difficult to interpret concentrations in relation to environmental stressors (Burgess *et al.*, 2017). However, combining this metric with faecal aldosterone concentrations (an indicator of adrenal activation) can allow the intrinsic and extrinsic factors affecting stress hormone levels to be better distinguished, allowing researchers to determine when populations are facing pressure from changes in their environment (Burgess *et al.*, 2017). We also encourage researchers to combine tools to gain a holistic snapshot of organismal response to environmental change, to better pin-point mechanisms underlying population declines, and for effective post-restoration monitoring. For instance, the incorporation of biologging and biotelemetry technology is already strengthening our understanding of the links between behaviour, movement, habitat selection, and energetic physiology and endocrinology for vulnerable aquatic and terrestrial species, with direct influences on conservation and management (Hussey *et al.*, 2015; Kays *et al.*, 2015; Wilson *et al.*, 2015). We anticipate this technology, in conjunction with measures of stress physiology, bioenergetics and nutritional physiology, will greatly expand our understanding of environmental tolerances, distribution and connectivity, and characteristics of optimal feeding and breeding habitats (Cooke, 2008). Indeed, current research suggests that when addressing conservation problems facing wild animals, it is impossible to consider physiology without also considering behaviour (Cooke *et al.*, 2013a).

Multi-trait assessments can also provide information on which components of the environment are influencing organisms of interest. For example, Ditmer *et al.* (2015) employed a modelling approach that could separate the influence of movement and habitat variables on heart rate in American black bears (*Ursus americanus*) allowing them to determine which aspects of the bears’ landscape (agriculture, roadways, fragmented forest) most influenced their physiology and behaviour. Further, Joly *et al.* (2015) combined assessments of diet composition, pregnancy rate, faecal hormones and sex ratios to provide a baseline of physiological and nutritional stress across life history stages and environmental qualities for barren-ground caribou (*Rangifer tarandus*) facing further human development in their range. In the future, this baseline will allow researchers to better pin-point how changes in food availability and human disturbance translate into changes in reproduction and fitness. Topics such as animal nutrition in a changing world inherently link physiology, ecology and health and demand multiple measures and endpoints (Birnie-Gauvin *et al.*, 2017).

Taking a multi-tool approach will best be informed by considering that environmental disturbances rarely occur in isolation. Organisms coping with human-induced change often experience multiple stressors, with alterations occurring in many components of environmental quality simultaneously (food availability and quality, predation risk, disease susceptibility, human presence, availability of shelter, etc.) (Breitburg *et al.*, 1998). Considering the ways in which a multi-stressor environment could influence physiology (Todgham and Stillman, 2013) can be enhanced by integrating knowledge of a study species’ natural and life history and this will help to determine the best tool combination in a given scenario. Of course, validations focused on determining which traits may best be measured in unison must also consider the cost of the tools. Measuring multiple hormonal traits (e.g. hormone panels) requiring lab assays and technician time may not be the most cost-effective approach for all conservation scenarios. From a practical perspective, the larger size of the plasma or faecal samples required, the greater laboratory time, and the higher costs associated with assays needed to assess multiple physiological traits simultaneously will (at least currently) make this approach much more applicable to large wildlife species and projects with greater scope. Nonetheless, we believe the push towards multi-trait point-of-care devices will greatly expand the ability of conservation physiologists to determine multiple components of stress and bioenergetic physiology in a cost-effective manner (Stoot *et al.*, 2014). Of 73 the papers we assessed, 26% employed multi-disciplinary approaches (i.e. they used tools from more than one physiological sub-discipline). When multi-trait assessments within the same sub-discipline are included (e.g. measuring multiple reproductive hormones), this percentage increases to 52%. As a result, we believe many researchers are beginning to appreciate the value of combining multiple traits when attempting to assess the ecological and conservation relevance of physiology.

### Build a comprehensive framework for plant conservation physiology

Figure 1 illustrates the small number of papers (3%) published in Conservation Physiology that have highlighted tool-based approaches for studying plant taxa. Yet, nearly half of all threatened and endangered species (47%) are found within the plant kingdom (IUCN, 2017). The limited breadth of the currently identified plant conservation physiology toolbox relative to the global challenge facing plant conservation efforts underscores the necessity to uncover, identify and validate cutting-edge tools. An obvious, but important question that was addressed in a previously published editorial in the journal (van Kleunen, 2014) is ‘what kind of plant studies fit within the scope of conservation physiology?’ The editorial identified three representative studies of plant conservation physiology: (i) phosphorus sensitivity of plants exposed to phosphite-containing fungicides (Lambers *et al.*, 2013); (ii) seed physiology in relation to storage, germination and growth conditions (Hay and Probert, 2013); and (iii) the physiological characteristics of non-native plants that successfully invaded low-resource environments (Funk, 2013). We suggest that in addition to these important studies and others like them, research should be targeted to identify physiological traits that underpin local adaptation to environmental stress, competition and disturbance.

Local adaptation is driven by divergent natural selection for traits that favour fitness in any given genotype–environment combination and should result in local populations that are more fit in their habitat compared to populations from other habitats (Kawecki and Ebert, 2004). In plants, the fitness traits that are largely selected for revolve around maintaining a favourable carbon budget. This in turn requires plants to fine-tune their carbon allocation strategy while simultaneously maximizing photosynthetic carbon gain given the limited availability of resources such as water, nutrients and sunlight (Long *et al.*, 2017). Allocation strategies that maximize growth and reproduction over other carbon sinks may be selected for in locations where fitness and survival are limited by competition. Conversely, locations where chronic stress and/or episodic disturbance outweighs competition, plants are likely selected to maximize labile carbon storage in the form of non-structural carbohydrates (NSC) over rapid growth. Traditionally, NSC storage was viewed as a consequence of weakening carbon sinks from reduced growth and respiration near the conclusion of the growing season when there is a surplus of carbon being acquired (Chapin *et al.*, 1990). Recent evidence, however, suggests that NSC storage may be highly regulated and is often a competing sink for recently acquired carbon throughout the growing season (Hoch *et al.*, 2002; Sala *et al.*, 2012). Thus, NSC concentrations (although somewhat laborious to measure) in plant tissues can be used to predict future mortality of genotypes, populations and species in response to episodic disturbance or chronic stress (Vilela *et al.*, 2016; Adams *et al.*, 2017). Robust information on plant labile carbon pools could in turn serve as a guide for prioritizing future restoration efforts, which are typically economically costly and challenging to implement (Long *et al.*, 2017).

Before plants can establish a carbon allocation strategy, they must first conduct photosynthesis to convert CO_2_ into sugars. In areas where climate change is increasing surface temperatures, plant tolerance to heat stress is governed largely by thermal regulation of photosynthetic tissues. Recent advances for measuring surface temperature using near remote sensing techniques, for example, are providing new avenues to evaluate heat stress and thermal regulation of plant canopies, leaves and other photosynthetic tissues. Leaf temperature measurements made in concert with other physiological and morphological leaf traits can yield important clues on likely plant responses to extreme thermal events. For example, measurements of plant transpiration, made directly on leaves or indirectly using stem sap flux sensors or remote sensing approaches can be used to quantify the evaporative cooling potential of individual plant leaves and canopies. Likewise, measurements of leaf morphology such as leaf size and specific leaf area can be used to evaluate radiative heat load and thermal capacitance of photosynthetic tissues. Improvements in fluorometers used to measure chlorophyll fluorescence—an analog of electron transport capacity during Photosystem II (Maxwell and Johnson, 2000)—are also facilitating new opportunities to comprehensively evaluate the potential performance of photosynthetic tissues under stress. Consequently, measurements of chlorophyll fluorescence, coupled with other leaf trait analyses are providing conservation physiologists with new metrics to evaluate plant stress responses to thermal extremes and other potential stressors (Arroyo-Pérez *et al.*, 2017; French *et al.*, 2017).

### Assess the linkages between environmental disturbance, physiology and fitness simultaneously

One of the most heavily proposed applications for physiology in conservation and management is to make inferences about wildlife disturbance based on either (i) a change in physiology from a previously established baseline or (ii) a difference in physiology between a reference population and a population facing an environmental change. There are two key validations necessary when verifying that a given physiological trait can be applied in this manner. First, the physiological metric must be sensitive to the environmental change of interest (Busch and Hayward, 2009), and the most robust test of this requires experimental manipulation of an environmental variable or condition while carefully controlling for other contexts that could influence physiological state (e.g. age, reproductive status, season, disease status, density). However, physiology changes regularly to allow organisms to cope with variation in their intrinsic and extrinsic environment without any negative consequences to the individual; therefore, witnessing a change in physiology is not sufficient to consider the metric a biomarker of disturbance. As a result, a second necessary validation involves ensuring the physiological trait shows a predictable relationship with fitness (Busch and Hayward, 2009; Madliger and Love, 2014). In that way, a change in physiology over time or a difference in average physiology between two populations can be used to anticipate a population-level response. Again, any tests of a physiology–fitness relationship must consider the multiple contexts that can influence physiology, fitness or the relationship between the two (Bonier *et al.*, 2009), or we risk the relationship no longer being applicable to conservation depending on the specific scenario.

While it has been common to test each of these validations separately, fewer investigations have simultaneously linked environmental quality, physiology, and fitness. However, this approach is essential to interpreting physiology as a relevant indicator of environmental disturbance (i.e. a metric that will signal individuals or populations are experiencing negative or positive effects due to a specific environmental alteration). Recently, Lea *et al.* (2018) completed this type of holistic validation in the vulnerable Cape mountain zebra (*Equus zebra zebra*). By linking faecal GC concentrations with both lower grass abundance (i.e. low habitat quality) and population growth rate, they were able to ensure monitoring of GCs will provide a biomarker of population viability, and that management interventions aimed at improving foraging habitat quality can also be assessed with this tool. More investigations are taking this approach (e.g. Kimball *et al.*, 2012; Kaiser *et al.*, 2015; Kleist *et al.*, 2018), and we encourage conservation physiologists to keep up this momentum to help identify which metrics, particularly those related to condition, stress, bioenergetics and immunity, will provide useful monitoring tools.

### Seize opportunities in other under-represented taxa

We presented a brief framework for plant conservation physiology above as we believe plants have been viewed by many to be outside the realm of conservation physiology until just recently (see van Kleunen, 2014). However, our investigation also indicated that invertebrates and amphibians have been under-represented in validation studies, compared to their relevance to the global biodiversity crisis. Conservation-relevant questions can indeed be addressed in these systems using physiology, and will require the same types of validations we have presented in other taxa. For example, non-invasively collected urinary metabolites of GCs and reproductive hormones have gained attention for monitoring reproductive status, health, and welfare in anurans (Narayan, 2013). To be applied in this way, validations such as whether the assays used to quantify them are detecting metabolites of interest, whether species-specific bacteria in the gut or metabolic rates influence detection, and how various temperatures and storage conditions may influence stability must be completed (see Narayan, 2013 for further discussion). Physiology has also been proposed as a useful tool in amphibians for understanding how host–parasite interactions will change in response to climate change scenarios (Rohr *et al.*, 2013). However, Rohr *et al.* (2013) outline a set of 14 questions that remain outstanding before we can have confidence in predictive models employing metabolic physiology in the context of disease ecology under climate change, indicating much validation is still necessary.

Invertebrates represent the most diverse collection of animals on earth, and physiological techniques in these organisms are relevant across a variety of conservation-related questions, particularly for insects. For example, trait-based ecological approaches in terrestrial invertebrates are revealing the effects of climate change on species distributions and how environmental stressors influence community assembly and distribution, as well as potentially offering tools for environmental risk assessment (e.g. of chemical contaminants) (Moretti *et al.*, 2017). There has been a recent push to standardize the measurement of physiological traits such as metabolic rate, growth rate, temperature tolerance and pH resistance, among others, and quantify responses and intra-specific variability using controlled experiments (Duffy *et al.*, 2015; Moretti *et al.*, 2017), which will also help to better apply them in conservation contexts. Recently, researchers have also begun to take a landscape physiology approach to understand the influence of habitat alteration on pollinator health (Alaux *et al.*, 2017). Continued investigation into how aspects of physiology (e.g. body mass, vitellogenin) and behaviour may be altered due to landscape composition will not only indicate how habitat changes are influencing pollinator persistence, but can also identify landscape management techniques that could provide essential habitat to bees and other pollinators (Alaux *et al.*, 2017). Finally, to address honey bee colony collapse, researchers are beginning to screen traits to identify markers that can predict abrupt depopulation of managed colonies, with some physiological metrics (e.g. vitellogenin) proving to have predictive power (Dainat *et al.*, 2012). As with many traits measured in vertebrates, the predictive value of this physiological trait has been shown to vary seasonally (Dainat *et al.*, 2012), and it will therefore be important to consider context-dependencies in the validation of other physiological traits in invertebrates. We reiterate to researchers that the field of conservation physiology is receptive to any work, regardless of scale and taxa, that enables physiological tools to be better applied to conservation endeavours and we encourage the publication of frameworks that outline the most pressing research questions and applications in under-represented taxa.

### Ensure validations are key components of research programmes

Of the 310 papers published from 2013 through 2018 in the journal, 24% are focused on some aspect of tool validation. This clearly illustrates the commitment many conservation physiologists are making to ensuring the techniques we have at our disposal are accurate and accessible. Even further, many of the papers (52%) we assessed here report instances where a tool faced a limitation (i.e. negative findings or complexity in interpretation of a physiological trait). This is reassuring since delineating these characteristics is equally as important as defining the instances where tools will best be applied. Publishing validations, methods-based papers, and negative findings is not always of high priority; however, we believe that Conservation Physiology’s Tool Box section is an indication that the field is highly receptive to these types of investigations. Just as there has been a growing re-appreciation for traditional natural history papers, we envision the same pattern occurring with tool-based papers for conservation physiology. To facilitate this, we can strive to continuously seek input from practitioners applying conservation physiology tools and techniques on the ground. Appreciating the logistical, monetary and interpretation limitations that are being faced is paramount to knowing which validations are most pressing. In addition, we can endeavour to have strong foundational knowledge of the role of the physiological variables we are measuring. By doing so, the potential applications of a given physiological tool can be delineated and tested most effectively. For example, in the case of hormones, knowing how they are regulated, their transport and binding mechanisms, whether they follow circadian rhythms, how they are metabolized and excreted, etc. provides information on the time periods a given measurement reflects, whether we can compare samples from different times of day, whether we are measuring a total or active portion of the circulating amount of hormone, etc. Often, investigations into these characteristics can be informative for both the applied and traditional arms of ecological and evolutionary physiology (Madliger and Love, 2015).

Moving forward, we implore all researchers in the realm of conservation physiology to include tool validations as part of their research programmes (Madliger, 2017); it is an integral component of contributing to a mission-oriented discipline. Indeed, it could be likened to the responsibility of participating in peer-review. Just as contributing time and effort to the review process ensures progress in the transfer of scientific knowledge, helping to validate tools within and across species, life history stages, environments, and demographic contexts, ensures that physiological techniques can meaningfully contribute to future success stories for conservation science. Ultimately, this is the goal of conservation physiology.

## Conclusion

The conservation physiology toolbox currently offers insight into immune function, stress, bioenergetics, cardiorespiratory physiology, toxicology, plant physiology, reproduction and sensory physiology. Although overall taxonomic coverage, contribution to conservation endeavours, biological scale of measurement and logistical options (e.g. level of invasiveness) are broadly represented, there is evidence of bias and the toolbox is still being actively validated in many capacities. However, we view this as a clear illustration that conservation physiologists are dedicated to ensuring their tools are as accurate and interpretable as possible. As traditional physiologists also develop field and laboratory techniques in their respective sub-disciplines, we know that conservation physiologists will continue to interpret and refine those tools in the light of conservation science. We look forward to seeing the toolbox grow and increase its applicability to more taxa, develop more non-invasive techniques, delineate where limitations exist, and identify the contexts necessary for interpretation in captivity and the wild.

## Supplementary Material

Supplementary Data
