# Supplementary material for: The conservation physiology toolbox: status and opportunities
Source: Conserv Physiol. 2018 Jun 19;6(1):coy029. doi: 10.1093/conphys/coy029 (PMC6007632; doi:10.1093/conphys/coy029)
Supplement: Supplementary Data [file coy029_supplementary_table_1_cons_phys_methods.docx]

| **Year**  **Supplementary Table 1.** List of tool-based papers published in *Conservation Physiology* from January 2013 through January 2018. | **Title** | **Author(s)** | **Article type** | **Physiological sub-discipline*** | **Taxa** |
| --- | --- | --- | --- | --- | --- |
| 2013 | Shotgun proteomics as a viable approach for biological discovery in the Pacific oyster | Timmins-Schiffman *et al.* | Tool Box | Genomics | Mollusc |
| 2013 | Development and evaluation of three mortality prediction indices for cold-stunned Kemp's ridley sea turtles (Lepidochelys kempii) | Stacy *et al.* | Research Article | Stress, Cardioresp | Reptile |
| 2013 | Effects of logging, hunting, and forest fragment size on physiological stress levels of two sympatric ateline primates in Colombia | Rimbach *et al.* | Research Article | Stress | Mammal |
| 2013 | Stress hormone concentration in Rocky Mountain populations of the American pika (Ochotona princeps) | Wilkening *et al.* | Research Article | Stress | Mammal |
| 2013 | Vulnerability of larval and juvenile white sturgeon to barotrauma: can they handle the pressure? | Brown *et al.* | Research Article | Cardioresp | Fish |
| 2013 | Sex, scarring, and stress: understanding seasonal costs in a cryptic marine mammal | Burgess *et al.* | Research Article | Stress, Bioenerg | Mammal |
| 2013 | Heart rate responses provide an objective evaluation of human disturbance stimuli in breeding birds | Ellenberg *et al.* | Research Article | Cardioresp | Bird |
| 2014 | A practical field extraction method for non-invasive monitoring of hormone activity in the black rhinoceros | Edwards *et al.* | Tool Box | Reproduc, Stress | Mammal |
| 2014 | Baleen hormones: a novel tool for retrospective assessment of stress and reproduction in bowhead whales (Balaena mysticetus) | Hunt *et al.* | Tool Box | Reproduc, Stress | Mammal |
| 2014 | Effects of natural environmental conditions on faecal glucocorticoid metabolite concentrations in jaguars (Panthera onca) in Belize | Mesa-Cruz *et al.* | Tool Box | Stress | Mammal |
| 2014 | Enzyme immunoassays as a method for quantifying hair reproductive hormones in two felid species | Terwissen *et al.* | Tool Box | Reproduc | Mammal |
| 2014 | Validation and use of hair cortisol as a measure of chronic stress in eastern chipmunks (Tamias striatus) | Mastromonaco *et al.* | Research Article | Stress | Mammal |
| 2014 | Validation of the i-STAT system for the analysis of blood parameters in fish | Harter *et al.* | Research Article | Stress, Cardioresp | Fish |
| 2014 | A novel technique to measure chronic levels of corticosterone in turtles living around a major roadway | Baxter-Gilbert *et al.* | Research Article | Stress | Reptile |
| 2014 | Quantifying long-term stress in brown bears with the hair cortisol concentration: a biomarker that may be confounded by rapid changes in response to capture and handling | Cattet *et al.* | Research Article | Stress | Mammal |
| 2014 | Evaluating physiological stress in Sumatran tigers (Panthera tigris ssp. sumatrae) managed in Australian zoos | Parnell *et al.* | Research Article | Stress | Mammal |
| 2014 | Non-lethal assessment of the reproductive status of broadnose sevengill sharks (Notorynchus cepedianus) to determine the significance of habitat use in coastal areas | Awruch *et al.* | Research Article | Reproduc | Elasmo |
| 2014 | Stress and reproductive hormones reflect inter-specific social and nutritional conditions mediated by resource availability in a bear–salmon system | Bryan *et al.* | Research Article | Stress, Reproduc | Mammal |
| 2015 | Developing a new research tool for use in free-ranging cetaceans: recovering cortisol from habour porpoise skin | Bechshoft *et al.* | Tool Box | Stress | Mammal |
| 2015 | Using novel methodologies to examine the impact of artificial light at night on the cortisol stress response in dispersing Atlantic salmon (Salmo salar L.) fry | Newman *et al.* | Tool Box | Stress | Fish |
| 2015 | Validation of the i-STAT and HemoCue systems for the analysis of blood parameters in the bar-headed goose, Anser indicus | Harter *et al.* | Research Article | Stress, Cardioresp | Bird |
| 2015 | Validation of the i-STAT system for the analysis of blood gases and acid–base status in juvenile sandbar shark (Carcharhinus plumbeus) | Harter *et al.* | Research Article | Stress, Cardioresp | Elasmo |
| 2015 | Optimization, validation and efficacy of the phytohaemagglutinin inflammation assay for use in ecoimmunological studies of amphibians | Clulow *et al.* | Research Article | Immunol | Amphibian |
| 2015 | Maximizing the reliability of non-invasive endocrine sampling in the tiger (Panthera tigris): environmental decay and intra-sample variation in faecal glucocorticoid metabolites | Parnell *et al.* | Research Article | Stress | Mammal |
| 2015 | The degradation of proteins in pinniped skeletal muscle: viability of post-mortem tissue in physiological research | Moore *et al.* | Research Article | Cardioresp | Mammal |
| 2015 | Faecal corticosterone metabolite concentrations are not a good predictor of habitat suitability for common gartersnakes | Halliday *et al.* | Research Article | Stress | Reptile |
| 2015 | Establishment of reference intervals for plasma protein electrophoresis in Indo-Pacific green sea turtles, Chelonia mydas | Flint *et al.* | Research Article | Immunol | Reptile |
| 2015 | Physiological predictors of long-term survival in juvenile Steller sea lions (Eumetopias jubatus) | Shuert *et al.* | Research Article | Immunol, Bioenerg | Mammal |
| 2015 | Blood gases, biochemistry and haematology of Galápagos marine iguanas (Amblyrhynchus cristatus) | Lewbart *et al.* | Research Article | Cardioresp, Stress, Bioenerg | Reptile |
| 2015 | Key metabolites in tissue extracts of Elliptio complanata identified using 1 H nuclear magnetic resonance spectroscopy | Hurley-Sanders *et al.* | Research Article | Bioenerg | Mollusc |
| 2015 | Oxidative stress predicts long-term resight probability and reproductive success in Scopoli's shearwater (Calonectris diomedea) | Constantini and Dell'Omo | Research Article | Stress | Bird |
| 2015 | Measurement of free glucocorticoids: quantifying corticosteroid-binding globulin binding affinity and its variation within and among mammalian species | Delehanty *et al.* | Research Article | Stress | Mammal |
| 2015 | Predicting future thermal habitat suitability of competing native and invasive fish species: from metabolic scope to oceanographic modelling | Marras *et al.* | Research Article | Bioenerg | Fish |
| 2015 | Detection of oocyte perivitelline membrane-bound sperm: a tool for avian collection management | Croyle *et al.* | Research Article | Reproduc | Bird |
| 2016 | Evaluating the effect of sample type on American alligator (Alligator mississippiensis) analyte values in a point-of-care blood analyser | Hamilton *et al.* | Tool Box | Bioenerg, Cardioresp, Stress | Reptile |
| 2016 | Development and application of an antibody-based protein microarray to assess physiological stress in grizzly bears (Ursus arctos) | Carlson *et al.* | Tool Box | Genomics, Stress | Mammal |
| 2016 | Calibration of the HemoCue point-of-care analyser for determining haemoglobin concentration in a lizard and a fish | Andrewartha *et al.* | Tool Box | Cardioresp | Reptile, Fish |
| 2016 | Longitudinal progesterone profiles in baleen from female North Atlantic right whales (Eubalaena glacialis) match known calving history | Hunt *et al.* | Tool Box | Reproduc | Mammal |
| 2016 | Methodological considerations for measuring glucocorticoid metabolites in feathers | Berk *et al.* | Tool Box | Stress | Bird |
| 2016 | Get the most out of blow hormones: validation of sampling materials, field storage and extraction techniques for whale respiratory vapour samples | Burgess *et al.* | Tool Box | Stress, Reproduc | Mammal |
| 2016 | Temporal overlap and repeatability of feather corticosterone levels: practical considerations for use as a biomarker | Harris *et al.* | Tool Box | Stress | Bird |
| 2016 | Effects of post-mortem storage conditions of bovine epididymides on sperm characteristics: investigating a tool for preservation of sperm from endangered species | Strand *et al.* | Tool Box | Reproduc | Mammal |
| 2016 | Employing individual measures of baseline glucocorticoids as population-level conservation biomarkers: considering within-individual variation in a breeding passerine | Madliger and Love | Research Article | Stress | Bird |
| 2016 | Validating faecal glucocorticoid metabolite analysis in the Virunga mountain gorilla using a natural biological stressor | Eckardt *et al.* | Research Article | Stress | Mammal |
| 2016 | Parasites, stress and reindeer: infection with abomasal nematodes is not associated with elevated glucocorticoid levels in hair or faeces | Carlsson *et al.* | Research Article | Stress, Immunol | Mammal |
| 2016 | Using circulating reproductive hormones for sex determination of Atlantic sturgeon (Acipenser oxyrinchus oxyrinchus) in the Saco River estuary, Maine | Wheeler *et al.* | Research Article | Reproduc | Fish |
| 2016 | Concentrations of faecal glucocorticoid metabolites in Asian elephant's dung are stable for up to 8 h in a tropical environment | Wong *et al.* | Research Article | Stress | Mammal |
| 2016 | Heavy with child? Pregnancy status and stable isotope ratios as determined from biopsies of humpback whales | Clark *et al.* | Research Article | Reproduc | Mammal |
| 2016 | Methods matter: considering locomotory mode and respirometry technique when estimating metabolic rates of fishes | Rummer *et al.* | Research Article | Bioenerg | Fish |
| 2016 | Sex-specific ecophysiological responses to environmental fluctuations of free-ranging Hermann's tortoises: implication for conservation | Sibeaux *et al.* | Research Article | Stress, Bioenerg, Cardioresp | Reptile |
| 2016 | Feather and faecal corticosterone concentrations predict future reproductive decisions in harlequin ducks (Histrionicus histrionicus) | Hansen *et al.* | Research Article | Stress | Bird |
| 2016 | Assessment of faecal glucocorticoid metabolite excretion in captive female fishing cats (Prionailurus viverinus) in Thailand | Khonmee *et al.* | Research Article | Stress | Mammal |
| 2016 | Physiological stress and post-release mortality of white marlin (Kajikia albida) caught in the United States recreational fishery | Schlenker *et al.* | Research Article | Stress | Fish |
| 2016 | Long-term effect of carbohydrate reserves on growth and reproduction of Prosopis denudans (Fabaceae): implications for conservation of woody perennials | Vilela *et al.* | Research Article | Plant | Plant |
| 2016 | Phenotypic variation in metabolism and morphology correlating with animal swimming activity in the wild: relevance for the OCLTT (oxygen- and capacity-limitation of thermal tolerance), allocation and performance models | Baktoft *et al.* | Research Article | Bioenerg | Fish |
| 2017 | An evaluation of the use of pentosidine as a biomarker for ageing turtles | Iverson *et al.* | Tool Box | Stress | Reptile |
| 2017 | Validation of a portable, waterproof blood pH analyser for elasmobranchs | Talwar *et al.* | Tool Box | Stress | Elasmo |
| 2017 | Obtaining accurate glucose measurements from wild animals under field conditions: comparing a hand held glucometer with a standard laboratory technique in grey seals | Bennett *et al.* | Tool Box | Bioenerg | Mammal |
| 2017 | Compatibility of preparatory procedures for the analysis of cortisol concentrations and stable isotope (δ13C, δ15N) ratios: a test on brown bear hair | Sergiel *et al.* | Tool Box | Stress | Mammal |
| 2017 | Development and testing of a simple field-based intermittent-flow respirometry system for riverine fishes | Mochnacz *et al.* | Tool Box | Bioenerg | Fish |
| 2017 | Multiple steroid and thyroid hormones detected in baleen from eight whale species | Hunt *et al.* | Tool Box | Stress, Reproduc | Mammal |
| 2017 | Semen collection and ejaculate characteristics of the Leopard Tortoise (Stigmochelys pardalis) | Zimmerman and Mitchell | Tool Box | Reproduc | Reptile |
| 2017 | Within-sample variation in snowshoe hare faecal glucocorticoid metabolite measurements | Lafferty *et al.* | Tool Box | Stress | Mammal |
| 2017 | The quantification of reproductive hormones in the hair of captive adult brown bears and their application as indicators of sex and reproductive state | Cattet et al. | Research Article | Reproduc | Mammal |
| 2017 | Qiviut cortisol in muskoxen as a potential tool for informing conservation strategies | Di Francesco *et al.* | Research Article | Stress | Mammal |
| 2017 | Blood gases, biochemistry and haematology of Galápagos hawksbill turtles (Eretmochelys imbricata) | Munoz-Perez *et al.* | Research Article | Cardioresp | Reptile |
| 2017 | Energy metabolism in mobile, wild-sampled sharks inferred by plasma lipids | Gallagher *et al.* | Research Article | Bioenerg | Elasmo |
| 2017 | Tools for the ex situ conservation of the threatened species, Cycladenia humilis var. Jonesii | Pence *et al.* | Research Article | Plant | Plant |
| 2017 | Molecular indices of viral disease development in wild migrating salmon | Miller *et al.* | Research Article | Genomics, Immunol | Fish |
| 2017 | Coupling gene-based and classic veterinary diagnostics improves interpretation of health and immune function in the Agassiz’s desert tortoise (Gopherus agassizii) | Drake *et al.* | Research Article | Genomics, Immunol | Reptile |
| 2017 | Corticosterone, inflammation, immune status and telomere length in frigatebird nestlings facing a severe herpesvirus infection | Sebastiano *et al.* | Research Article | Stress, Immunol | Bird |
| 2018 | Monitoring ovarian cycles, pregnancy and post-partum in captive marsh deer (Blastocerus dichotomus) by measuring fecal steroids | Polegato *et al.* | Research Article | Reproduc | Mammal |
| 2018 | Impact of gas emboli and hyperbaric treatment on respiratory function of loggerhead sea turtles (Caretta caretta) | Portugues *et al.* | Research Article | Cardioresp | Reptile |

*bioenerg = bioenergetics; cardioresp = cardiorespiratory physiology; genomics = genomics & proteomics; immunol = immunology & epidemiology; plant = environmental plant physiology; reproduc = reproductive physiology; stress = stress physiology
